# Key performance indicators of offensive transitions in elite women’s football: a machine learning and explainability approach

**DOI:** 10.5114/biolsport.2026.153309

**Published:** 2025-08-05

**Authors:** Claudio A. Casal, José Luis Losada, Ana M. de Benito Trigueros, Rubén Maneiro, Iyán Iván-Baragaño

**Affiliations:** 1 Faculty of Physical Education & Sport Sciences, Catholic University of Valencia, San Vicente Mártir, Valencia, Spain; 2 Department of Social Psychology and Quantitative Psychology, University of Barcelona, Barcelona, Spain; 3 HeQoL Research Group. Department of Physical and Sport Education, University of León, León, Spain; 4 Faculty of Education and Sport, University of Vigo, Vigo, Spain; 5 Department of Sports Sciences, Faculty of Medicine, Health and Sports, European University of Madrid, Madrid, Spain

**Keywords:** Women’s soccer, Observational methodology, Match observer, Performance analysis, Dynamic offensive transitions

## Abstract

Women’s football has experienced significant growth in sporting, economic, and social interest. However, there remains a shortage of studies examining key technical-tactical performance indicators in elite competitions. This research aimed to identify and quantify the influence of technical-tactical indicators on the effectiveness of offensive dynamic transitions in elite women’s football, using a machine learning approach. To this end, 3,610 dynamic offensive transitions recorded across 35 matches from the final stages of the FIFA Women’s World Cup 2023, UEFA Women’s Euro, and UEFA Women’s Champions League 2023/24 were analysed using an observational methodology. An *ad hoc* observation instrument, *Transfootb*, was developed to record teams’ offensive behaviours, opponents’ defensive responses, and the match context at the time of the offensive dynamic transition. A chi-square test was applied to identify associations between variables, followed by the training of a Random Forest model to predict transition outcomes. Additionally, ShAP values were computed and visualised to interpret the influence of the predictors. The model achieved an area under the curve of 0.78, with a recall of 18% and a specificity of 96%. The results indicate that the execution mode of offensive transitions and the match context significantly influence offensive success. Specifically, penetrating passes (≥ 3), counterattacks, and an opponent’s low defensive positioning were the key predictors of successful offensive transitions. This study provides valuable scientific evidence to optimise strategic decision-making in dynamic offensive transitions in elite women’s football. Furthermore, it highlights the potential of machine learning in analysing and predicting performance in sport-specific actions.

## INTRODUCTION

Interest in women’s football is experiencing unprecedented growth. According to the latest FIFA report [1], the number of players participating in organised football has increased by 24% over the past four years, reaching 16 million worldwide [1]. In certain cases, such as Spain, this growth has been even more remarkable, with the number of registered female players doubling over the same period [2] and, for the first time in history, exceeding 100,000 federated licences. Moreover, the media, economic, and sporting impact of the World Cup has played a pivotal role in this expansion, generating an economic impact of $1.32 billion in Australia alone [3].

In the scientific domain, while women’s football has been explored from various perspectives – including those of players, fans, and sports organisations [4] – a significant research gap remains in studies focused exclusively on the women’s game [5]. Some authors have highlighted the limited attention and information available regarding key determinants in women’s football, such as match analysis [6]. However, although research in this area began later than in the men’s game – particularly in fields such as match analysis [7]-, academic publications have nonetheless achieved significant impact within the research community.

Technical and tactical performance analysis has evolved significantly since 2020, driven by the increasing availability of data from various technological sources. A key example is the FIFA’s use of optical tracking, which has enhanced understanding of the relationship between team level and conditional performance [8, 9], as well as its application in tactical analysis through deep learning techniques [10]. Similarly, access to event data from high-level women’s competitions has facilitated studies such as that of Trower et al. [11], which identified 10 distinct player profiles based on the analysis of recorded events across five consecutive seasons of the Women’s Super League (WSL). Likewise, Narayanan & Pifer [12] conducted a comparative study of the U.S. women’s national team and its rivals using data provided by *Statsbomb* [https://statsbomb.com/es/]. Their findings indicated that the technical performance of the American team exceeded that of its opponents in key indicators such as shots on target and successful dribbles.

At the collective level, various investigations have examined the relationship between tactical indicators and team success – both globally and partially (i.e., based on match outcomes or possession outcomes, respectively) – as well as their association with game strategies. A notable example is the study of Iván-Baragaño et al. [13], which developed a multivariate model combining tactical indicators to estimate the probability of success in a possession. Regarding the contextual variables, Harkness-Armstrong et al. [14] analysed approximately 200 youth players in England and found that the phase of play (in-possession or out-of-possession), in combination with match status, significantly modified players’ conditional demands. Likewise, match status was identified as a key factor influencing team’s offensive strategies during the FIFA Women’s World Cup 2015 (FWWC, 2015) [15]. However, a subsequent study on the FWWC 2019 found that teams maintained consistent offensive strategies and success rates in their attacking play, regardless of match status [16].

On the other hand, studies such as that of Martínez-Hernández et al. [17], which used the WSL as a sample, analysed movement patterns preceding conceded goals, identifying the most frequent as linear advancing motion, deceleration, or turning, among others. In any case, it is evident that the two competitions that have generated the greatest research interest regarding technical-tactical indicators in women’s football have been FWWC 2019 and 2023. In relation to these tournaments, studies such as both conducted by Bradley [8, 9] provided a detailed analysis of conditional demands based on both player position and team characteristics. Their findings highlighted differences in physical demands according to positional roles and the quality of opposition, aligning with evidence presented a decade earlier by Hewitt et al. [18].

Other studies have focused on evaluating the influence of match outcomes and tournament stages in the FWWC 2023 [19], as well as comparing performance across confederations in the same edition. In this regard, Ju et al. [20] found that the teams from the Union of European Football Associations (UEFA) in the FWWC 2023 demonstrated superior technical performance in key metrics such as pass completion percentage. Meanwhile, variability in highintensity distances covered was greater among teams from the Confederation of African Football (CAF) compared to those from other confederations.

Finally, one of the primary challenges facing women’s football remains the disparity between domestic leagues [21], a factor that directly influences spectator interest in the sport. In this context, studies such as that of Casal et al. [22], which focus on analysing a single league, provide valuable insights into the game strategies employed within specific competition.

Regarding the development of predictive models capable of classifying event outcomes, a key challenge is class imbalance, particularly when dealing with low-frequency events. In football research, various studies have attempted to predict possession outcomes [23, 24] or the occurrence of injuries [25, 26], predominantly using binary classification models. In many cases, the event of interest has a low relative frequency, which negatively impacts the model’s ability to predict positive outputs (recall). This challenge arises from the difficulty algorithms face in identifying consistent patterns within the selected training features. To mitigate this limitation, oversampling techniques have been developed [27, 28], generating synthetic samples from the original dataset to enhance the model’s predictive capacity.

This research pursues three main objectives: (i) to identify the technical-tactical indicators associated with goals and shots during dynamic offensive transitions in elite women’s football; (ii) to train a supervised machine learning model, optimized through hyperparameter tuning, to predict possession outcomes; and (iii) to analyse the influence of each criterion on the model’s output using extrinsic explainability techniques.

To maximise the external validity of the findings, three international competitions – both at the club and national team levels – were analysed, as they are considered among the most prestigious tournaments worldwide. As a novel contribution, statistical and machine learning techniques – such as oversampling – were applied, which remain largely unexplored in the context of women’s football.

## MATERIALS AND METHODS

### Design and participants

This research work was developed within the framework of observational methodology [29], employing the following design: nomothetic, though the analysis of the performance of 3,610 dynamic offensive transitions executed by various teams; punctual intra-sessional, the dynamic offensive transitions corresponding to different matches, and multi-dimensional because we analysed the multiple dimensions that constituted the *ad hoc* instrument used, formed by a system of categories and field formats [30].

In order to control for situational variables that may influence teams’ tactical and strategic behaviours – such as opponent quality or match status – a total of 35 matches from the final stages of the FWWC 2023 (n = 1,535), UEFA Women’s Euro 2022 (n = 750) and UEFA Women’s Champions League 2023/24 (n = 1,325) were selected. Across these matches, 3,610 dynamic offensive transitions were analysed, following the exclusion of 119 transitions due to lack of observability. Match recordings were obtained from WyScout (www.hudl.com, s.f.) and analysed post-event. Matches were observed for the regular period (i.e. 90 min, excluding extra time). The recording of the information was carried out respecting the spontaneity of the players’ behaviour and in their natural environment. According to the Belmont Report [31], the use of public images for research purposes does not require informed consent or approval by an ethics committee.

### Observation and recording instrument

The pillars on which the construction of the observation instrument has been based were the following: (i) a previous theoretical framework; (ii) criteria and categories collected empirically in other observational studies; (iii) and, finally, novel criteria that were tested in this work. The methodological steps implemented were as follows: First, the problem was identified, and a scientific group of experts was formed, composed of two academics (with PhDs in Physical Activity and Sport Sciences) and UEFA PRO coaches, with more than ten years of experience in observational methodology and performance analysis in football. After consulting the empirical evidence and based on the defined objectives, a first selection of the instrument’s tactical indicators was made, and a first exploratory post-event observation was carried out. Subsequently, after a discussion by the expert group, the instrument was readjusted, and another post-event observation was carried out. This process was repeated four times until finally creating the observation instrument *ad hoc*, called *Transfootb* (Table 1), consisting of a total of 18 criteria in which information is included regarding contextual variables, start, development and completion of the offensive phase of the observed team and how to execute the defensive transitions of the opposing team.

**TABLE 1:** Criteria, category and codes to observational instrument, *Transfootb*

	Criteria	Category	Code
Contextual variables	Tournament (TN)	**FWWC 2023:** FIFA World Women Cup	FWWC
		**Women’s EURO 2022**	EURO
		**UWCL 2023/24:** UEFA Women Champions League	UWCL

	Stage (ST)	**Round of 16**	R16
		**Quarter-final**	QF
		**Semi-final**	SF
		**Final**	FF

	Period of Match (T)	**0–15 Minutes:** 0–15 minutes of the match time	0–15
		**16–30 Minutes:** 16–30 minutes of the match time	16–30
		**31–45 Minutes:** 31 minutes – half time	31–45
		**46–60 Minutes:** 46–60 minutes of the match time	46–60
		**61–75 Minutes:** 61–75 minutes of the match time	61–75
		**76–90 Minutes:** 76 minutes – full time	76–90

	Match Status (MS)	**Winning:** The observed team has scored more goals than the opposing team at the moment of regaining possession of the ball	WN
		**Drawing:** The observed team has scored equal goals to the opposing team, or no goals had been scored	DR
		**Losing:** The observed team has scored less goals than the opposing team at the moment of regaining possession of the ball	LS

	Match Outcome (MO)	**Win:** The observed team has scored more goals than opponent and won the match	FW
		**Draw:** The observed team has scored equal goals to opponent and draw the match	FD
		**Loss:** The observed team has scored fewer goals than opponent and lost the match	FL

Dynamic offensive transition	Ball Recovery (BR)	**Steal:** A defending player prevents the ball passed by an opponent from reaching its intended receiver by contacting the ball and maintaining his team’s possession of the ball	ST
		**Duel:** A defending player dispossesses an opponent of the ball through a physical challenge or defensive pressure	DL
		**Turnover:** A defending player collects the ball lost (via clearance or a missed pass) by the opposing team	TR
		**Goalkeeper Action:** The goalkeeper recovers the ball after an opponent’s shot, cross, turnover, etc.	GK

	Recovery Zone (RZ) (Fig. 1)	**Defensive zone**	DF
		**Middle defensive zone**	MD
		**Middle offensive zone**	MO
		**Offensive zone**	OF

	Start Interaction Context (CEI)	The goalkeeper regains possession of the ball with the opposing team’s forward line ahead	PA
		The defensive line regains possession of the ball with the forward line ahead	RA
		The defensive line regains possession of the ball with the midfield line ahead	RM
		The midfield line regains possession of the ball against the rearmost line	MR
		The midfield line regains possession of the ball against the midfield line	MM
		The midfield line regains possession of the ball with the forward line ahead	MA
		The forward line regains possession of the ball against the rearmost line	AR
		The forward line regains possession of the ball against the midfield line	AM
		The forward line regains possession of the ball against the goalkeeper	AØ

	Type of Initial Attack (TA)	**Positional:** Possession starts by gaining the ball in play; the first or second player makes short, horizontal, and non-penetrating passes in an attempt to destabilize the organized defensive system of the opposing team	PT
		**Direct:** Possession starts by gaining the ball in play; the first or second player in action uses long vertical penetrating passes. This type of possession aims to quickly reach the opponent’s goal, challenging the organized defensive system of the opposing team	DT
		**Counterattack:** Possession starts by gaining the ball in play; the first or second player in action uses penetrating passes or dribbles to penetrate; the progression towards the opposing goal involves a high percentage of quick penetration passes (evaluated qualitatively). This type of possession aims to deny the opponent the opportunity to minimize surprise, reorganize their system, and be defensively prepared. It cannot begin with a goalkeeper pass if the goalkeeper controls the ball for more than 4 seconds	CT

	Passes (PS)	**0:** The attacking team fails to make any passes	0
		**1–2:** The attacking team makes between 1 and 2 passes	1–2
		**3–4:** The attacking team makes between 3 and 4 passes	3–4
		≥ **5**: The attacking team makes 5 or more passes	≥ 5

	Penetrative Passes (PP)	**0:** The team does not make any passes towards the opposing goal, failing to surpass any player or defensive line of the opposing team	0
		**1–2:** The team makes between 1 or 2 passes towards the opposing goal, successfully surpassing some player or defensive lines of the opposing team	1–2
		≥ **3:** The team makes more than 2 passes towards the opposing goal, successfully surpassing some players or defensive lines of the opposing team	≥ *3*

	Attack Player (AP)	**1–2:** During the team’s possession, between 1 and 2 players voluntarily contact the ball If a player contacts the ball more than once, it is counted only once	1–2
		**3–4:** During the team’s possession, between 3 and 4 players voluntarily contact the ball	3–4
		≥ **5:** During the team’s possession, 5 or more players voluntarily contact the ball	≥ 5

	End Zone (EZ) (Fig. 1)	**Defensive zone**	DFF
		**Middle defensive zone**	MDF
		**Middle offensive zone**	MOF
		**Offensive zone**	OFF

	Type of Possession (TP)	**Short possession:** one or two passes per team possession	SH
		**Medium possession:** three or four passes per team possession	MP
		**Long possession:** five or more passes per team possession	LG

Dynamic defensive transition opposing team	General Defensive Approach (PTGD)	**Persistent (pressure):** Several opposing players press the attackers during the first 3 seconds of possession. The defenders position themselves near the ball possessor, trying to hinder their actions, and close to the attackers closest to the ball, attempting to prevent passes. Pressing defensive model.	PR
		**Expectant (no pressure):** A player pressures the ball possessor, or no player pressures the attackers during the first 3 seconds of possession. Containment defensive model.	EP

	Number of Defenders (DOP)	**1–3:** At the moment of regaining possession, the opposing team has between 1 and 3 players positioned between the ball and their own goal, excluding the goalkeeper.	1–3
		**4–6:** At the moment of regaining possession, the opposing team has between 4 and 6 players positioned between the ball and their own goal, excluding goalkeeper.	4–6
		≥ **7:** At the moment of regaining possession, the opposing team has 7 or more players positioned between the ball and their own goal, excluding goalkeeper.	≥ 7

	Defensive Position (POT)	**High:** The furthest-back opponent is in the opposing half	HG
		**Medium:** The furthest-back opponent is closer to the midline than to their own goal (Middle def zone)	ME
		**Low:** The furthest-back opponent is closer to their own goal than the midline (Def zone)	LW

Transition Outcome	Outcome (OUT)	**Goal:** When the whole of the ball crosses over the line, between the goal posts and under the crossbar, provided no offence has been committed by the scoring team. The referee awarded a goal	GO
		**Attempt ON Target:** An attempt on goal by the attacking team that were heading towards the goal which was saved by the goalkeeper or blocked by a defensive player of the opposing team	AO
		**Attempt OFF Target:** An attempt by the attacking team which was not directed between the dimensions of the goal including hitting the crossbar or goal posts	AF
		**Set-play:** A set piece was awarded to the attacking team in the form of a free kick, penalty kick or throw-in	SP
		**Corner kick:** The attacking team wins a corner kick	CK
		**Enter offensive zone:** advance the ball into the offensive zone, free kicks, and throw-ins in the offensive zone.	OZ
		**Loss of Possession:** The attacking team lost possession of the ball through the ball going out of the dimensions of the pitch or an opposing team player regaining possession of the ball, with enough control to have a deliberate influence over the ball’s subsequent direction	LP

### Procedure and reliability

Data were coded by one observer and, prior to the coding process, to reduce intra-observer variability, ten training sessions were carried out in which 250 transitions, not included in the final sample, were coded. The criterion of consensual agreement [32] among the observer and the principal investigator, so that recording was only done when agreement was produced. Cohen’s Kappa coefficient was calculated to intra and inter-observer reliability test, through reassessment of 361 offensive transitions (10%) randomly selected, four weeks after the initial analysis. Reliability of each criteria is presented in Table 2, with general defensive approach opposing team presenting the lowest value (0.89, 0.84), considered *almost perfect* according to Fleiss et al. [33] scale.

**TABLE 2:** Intra and inter-observer agreement

Criteria	Kappa coefficient	

	Intra-observer	Inter-observer
Period of Match	1.00	1.00
Match Status	1.00	1.00
Match Outcome	1.00	1.00
Ball Recovery	0.98	0.95
Recovery Zone	1.00	1.00
Start Interaction	0.95	0.90
General Defensive Approach Opposing Team	0.89	0.84
Number of Defenders Opposing Team	1.00	0.98
Defensive Position Opposing Team	1.00	1.00
Type of Initial Attack	0.97	0.89
Passes	1.00	1.00
Penetrative Passes	0.94	0.91
Attack Player	1.00	1.00
End Zone	1.00	1.00
Type of Possession	1.00	1.00
Possession Outcome	1.00	1.00

### Statistical analysis

First, a bivariate analysis was carried out using contingency tables and the analysis of absolute and relative frequencies. The existence of a statistically significant association between the analysed criteria was quantified from the statistic χ2 (p < .05). For the criteria that showed association, the effect size was calculated from the Contingency Coefficient, considered as small (ES = .10), medium (ES = .30) and large (ES = .50) [34].

Next, a Random Forest [35] supervised model was trained in which all the predictor variables were included with the exception of tournament, stage and end zone, a decision made with the aim of avoiding collinearity problems and increasing the generalizability of the results. To do this, the Outcome variable was mapped to a binary variable creating the categories Success (Goal, Attempt on target, Attempt off Target) and No Success (Rest of categories of the Outcome variable), this decision is justified in order to obtain a greater predictive capacity regarding the binary problem, rather than a multiclass problem. Once mapped and due to the class imbalance of the variable to be predicted (Success = 10.4% – No Success = 89.6%), an oversampling of the unbalanced category was carried out using the SMOTE technique. Although the use of this technique may lead to overfitting in the training dataset, it was considered essential to increase the model’s recall, because previous work has shown an improvement in the classification of the models [27]. The trained Random Forest model, based on the random assembly of decision trees, has been used in other previous studies with classification objectives in the field of sport [36, 37] due to its high predictive capacity. For its training, the resampled dataset was segmented in a stratified way based on the target variable in 70% training and 30% testing. In addition, different combinations of hyperparameters were tested using a cross validation procedure with 5 folds in the training sample. The predictive performance of the model was evaluated from the classification matrix, both on the resampled dataset and on the original dataset, as well as by calculating the area under the curve (AUC), considered as excellent (0.90 < AUC < 1.00), good (0.80 < AUC < 0.90), fair (0.70 < AUC < 0.80), poor (0.60 < AUC < 0.70), and fail (0.50 < AUC < 0.60) [38].

Finally, the explainability technique ShAP [39] was implemented on each category analysed. The technique consists of the calculation of the Shapley Additive exPlanation values. This approach quantifies the contribution of each category by integrating all variables based on their expected value in the model output, following the formula, allowing to attribute to each analysed variable the change on the prediction of the model, an aspect that allows to perform an interpretability of black box models such as Random Forest. For this reason, other authors have applied this technique in applied fields such as data analysis in football [40, 41].

## RESULTS

Bivariate results are presented in Table 3. A total of 13 criteria showed a statistically significant association with the mapped Outcome variable.

**TABLE 3:** Bivariate analysis between the dependent criterion Possession Outcome and the other criteria analysed.

Criteria	Categories	Success (n = 375, 10.4%)	No success (n = 3,235, 89.6%)	p-value [ES]
Tournament	FWWC 2023	139 (37.1%)	1,396 (43.2%)	p > .05 [-]
	Women´s Euro 2022	72 (19.2%)	678 (20.9%)	
	UWCL 2023/24	164 (43.7%)	1,161 (35.9%)	

Stage	Final	35 (9.3%)	224 (6.9%)	p > .05 [-]
	Semifinal	76 (20.3%)	686 (21.2%)	
	Quarter Final	186 (49.6%)	1,547 (47.8%)	
	Round of 16	78 (20.8%)	778 (24%)	

Period of Match	1–15	58 (15.5%)	589 (18.2%)	p > .05 [-]
	16–30	59 (15.7%)	457 (14.1%)	
	31–45	53 (14.1)	523 (16.2%)	
	46–60	58 (15.5%)	552 (17.1%)	
	61–75	64 (17.1%)	466 (14.4%)	
	76–90	83 (22.1%)	648 (20.0%)	

Match Status	Drawing	177 (47.2%)	1755 (54.3%)	p < .005 [.05]
	Losing	89 (23.7%)	749 (23.2%)	
	Winning	109 (29.1%)	731 (22.6%)	

Match Outcome	Win	181 (48.3%)	1,211 (37.4%)	p < .001 [.07]
	Draw	89 (23.7%)	844 (26.1%)	
	Loss	105 (28.0%)	1,180 (36.5%)	

Ball Recovery	Steal	107 (28.5%)	918 (28.4%)	p < .05 [.05]
	Duel	80 (21.3%)	584 (18.1%)	
	Turnover	168 (44.8%)	1,402 (43.3%)	
	Goalkeeper Action	20 (5.3%)	331 (10.2%)	

Recovery Zone	Defensive Zone	84 (22.4%)	1,307 (40.4%)	p < .05 [.20]
	Middle Defensive Zone	109 (29.1%)	1,127 (34.8%)	
	Middle Offensive Zone	132 (35.2%)	715 (22.1%)	
	Offensive Zone	50 (13.3%)	86 (2.7%)	

Start Interaction Context	PA	21 (5.6%)	335 (10.4%)	p < .001 [.19]
	RA	117 (31.2%)	1,500 (46.4%)	
	RM	4 (1.1%)	23 (0.7%)	
	MR	2 (0.5%)	17 (0.5%)	
	MM	181 (48.3%)	1,212 (37.5%)	
	MA	2 (0.5%)	53 (1.6%)	
	AR	36 (9.6%)	67 (2.1%)	
	AM	9 (2.4%)	28 (0.9%)	
	AØ	3 (0.8%)	0 (0.0%)	

General Defensive	Persistent (Pressure)	166 (44.3%)	1,467 (45.3%)	p > .05 [-]
Approach Opposing Team	Expectant (No Pressure)	209 (55.7%)	1,768 (54.7%)	

Number of Defenders Opposing Team	1–3	35 (9.3%)	81 (2.5%)	p < .001 [.13]
	4–6	93 (24.8%)	545 (16.8%)	
	≥ 7	247 (65.9%)	2,609 (80.6%)	

Defensive Position Opposing Team	High	72 (19.2%)	1,170 (36.2%)	p < .001 [.14]
	Medium	105 (28.0%	1,039 (32.1%)	
	Low	198 (52.8%)	1,026 (31.7%)	

Type of Initial Attack	Positional Attack	94 (25.1%)	1,347 (41.6%)	p < .001 [.17]
	Direct Attack	175 (46.7%)	1,546 (47.8%)	
	Counterattack	106 (28.0%)	342 (10.6%)	

Passes	0	29 (7.7%)	436 (13.5%)	p < .001 [.10]
	1–2	117 (31.2%)	1,316 (40.7%)	
	3–4	87 (23.2%)	639 (19.8%)	
	≥ 5	142 (37.9%)	844 (26.1%)	

Penetrative Passes	0	38 (10.1%)	1,109 (34.3%)	p < .001 [.25]
	1–2	206 (54.9%)	1,812 (56.0%)	
	≥ 3	131 (34.9%)	314 (9.7%)	

Attack Player	1–2	78 (20.8%)	1,206 (37.3%)	p < .001 [.12]
	3–4	148 (39.5%)	1,218 (37.7%)	
	≥ 5	149 (39.7%)	811 (25.1%)	

End Zone	Defensive Zone	0 (0.0%)	176 (5.4%)	p < .001 [.42]
	Middle Defensive Zone	2 (0.5%)	821 (25.4%)	
	Middle Offensive Zone	8 (2.1%)	1,391 (43%)	
	Offensive Zone	365 (97.3%)	847 (26.2%)	

Type of Possession	Short Possession	46 (12.3%)	759 (23.5%)	p < .001 [.10]
	Medium Possession	75 (20.0%)	805 (24.9%)	
	Long Possession	254 (67.7%)	1,671 (51.7%)	

In relation to the trained Random Forest model, the combination of hyperparameters with higher performance obtained through the cross validation procedure was: ‘bootstrap’: False, ‘max_depth’: 20, ‘min_samples_leaf’: 2, ‘min_samples_split’: 2, ‘n_estimators’: 300. The area under the curve over the resampled dataset was excellent (AUC = .99) and fair (AUC = .78) over the original set. The confusion matrices are presented in Figure 2 and the different evaluation metrics of both models (original and resampled test set) in Table 4. Overall, the model presented a high overall classification capacity (accuracy), with a correct classification percentage of 95% in the test set over the resampled dataset and 88% over the test set in the original dataset. In contrast, when correctly classifying the positive output (recall) the performance of the model decreased significantly in the original dataset (18%) compared to the resampled dataset (94%), while the true negative rate (specificity) was 95% and 96% for the resampled and original test sets, respectively. Results highlight substantial improvements in recall following resampling, while maintaining high levels of accuracy and specificity.

**FIG. 1:**
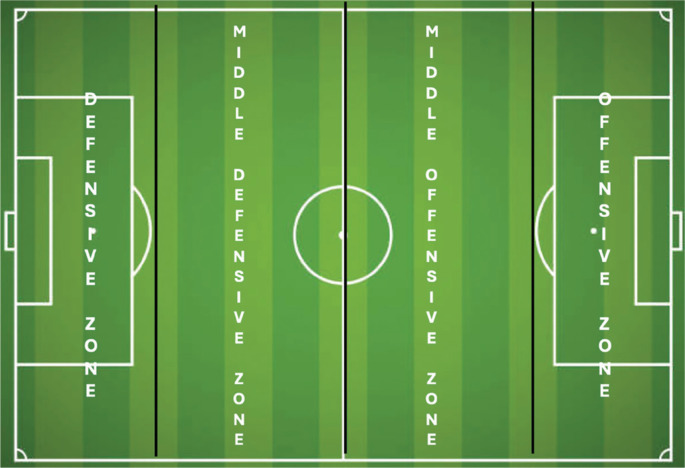
Spatial division of the football pitch.

**FIG. 2:**
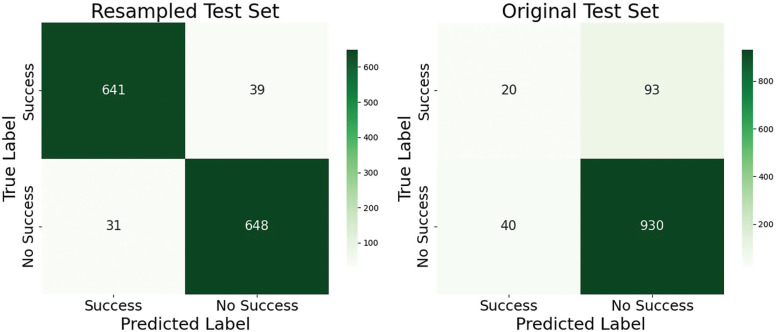
Confusion matrices of the Random Forest model over the resampled and original dataset.

**TABLE 4:** Performance metrics for the success prediction model evaluated on the resampled and original test sets.

	Success Prediction	

	Original Test set	Resampled Test set
Accuracy	0.88	0.95
Recall	0.18	0.94
Specificity	0.96	0.95

Results highlight substantial improvements in recall following resampling, while maintaining high levels of accuracy and specificity.

**FIG. 3:**
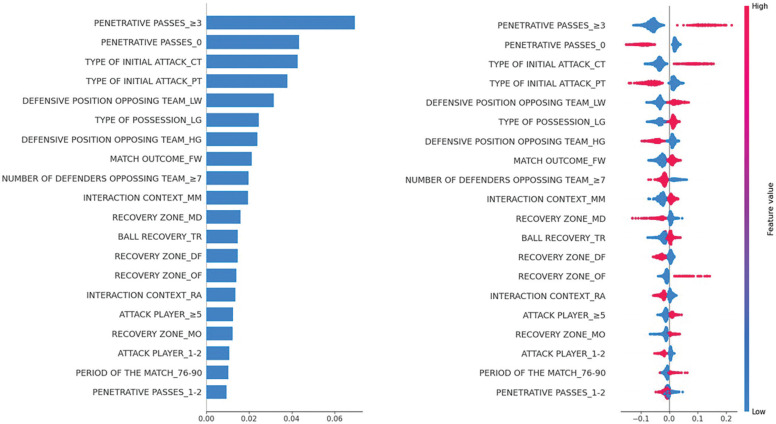
Influence of the analysed criteria and categories on the model’s output. ‘Note: The red-pink colours to the right of the value 0 on the vertical axis indicate that the presence of the corresponding category increases the likelihood that the model will predict the positive category of the target variable.

Regarding the influence of different criteria and categories on the binary classification model, Penetrative Passes emerged as the most impactful criterion, increasing the likelihood of the model predicting Success when its value reached ≥ 3. The second most influential criterion was Type of Initial Attack, with a higher probability of a positive output when the category corresponded to Counterattack (CT). Similarly, Defensive Position of the Opposing Team affected the probability of success when classified as Low, as did Type of Possession when it corresponded to Long. The influence of each criterion and its respective categories is illustrated in Figure 3, which ranks them in descending order based on their impact on the model’s classification.

## DISCUSSION

This study aimed to identify key performance indicators (KPIs) associated with offensive success in dynamic offensive transitions in elite women’s football, develop a predictive model for offensive success, and evaluate the influence of these KPIs on the model’s outcome. To enhance the applicability of the findings, an extrinsic explainability technique was employed, enabling a clearer understanding of the impact of each variable on the model.

A total of 3,729 dynamic offensive transitions were recorded across 35 matches, averaging 106.5 transitions per match – consistent with data reported in men’s football by Casal et al. [42]. Of the 3,610 transitions analysed, only 1.3% resulted in a goal, 9.1% in attempt, 21.1% in an entry into the offensive zone, while 48.9% led to possession loss. These results, aligning with previous studies such as that of Iván-Baragaño et al. [22], highlight the relatively low offensive effectiveness of the transitions examined.

Among all indicators analysed, the number of penetrating passes exhibited the strongest relationship with outcome. Specifically, performing three or more penetrating passes had a greater positive influence on success, whereas making none or only one or two was more frequently associated with no success outcomes. As shown in Table 3, 34.9% of possession involving three or more penetrating passes resulted in success, compared to only 9.5% of those that did not. Similar patterns have been observed in men’s football, with studies by Tenga et al. [43] and Zani et al. [44] demonstrating that teams executing a higher number of penetrating passes are more likely to create goal-scoring opportunities. This trend may be explained by the disruptive effect of these passes on the opposing team’s defensive structure, as they facilitate disorganisation, hinder defensive reactions, and allow the ball to be received in more advanced areas with reduced defensive pressure.

The counterattack, as a type of initial attack following ball recovery, was the second most influential indicator in relation to outcome, achieving an effectiveness rate of 28.0% compared to 10.6% for other types of attack. Conversely, positional attacks proved less effective, with a success rate of 25.1% compared to a failure rate of 41.6%. These results reinforce the notion that, in dynamic offensive transitions, capitalising on the opponent’s defensive imbalance immediately after regaining possession is crucial for creating goal-scoring opportunities.

The defensive position opposing team was the third most significant indicator linked to outcome. Specifically, a low defensive position was associated with higher offensive effectiveness, whereas a high defensive position reduced attacking efficiency. This relationship may be explained by the statistical association between defensive positioning and the recovery zone (p < .001). The closer to the opponent’s goal possession is regained, the deeper the opposing defensive line tends to be. Figure 3 illustrates that ball recoveries in advanced zones (OF, MO) promote offensive success, whereas recoveries in deeper zones (DF, MD) hinder it. These results align with previous research, such as Iván-Baragaño et al. [22] and Scanlan et al. [45], which also establish a link between recovery zones and offensive success. Although Scanlan et al. [45] employed a different field segmentation, their findings similarly highlight the middle offensive and offensive zones as the most effective, further supporting our conclusions.

Additionally, the start interaction context demonstrated a strong relationship with the aforementioned indicators and significantly influenced outcomes. Specifically, the MM category was associated with success, whereas RA was linked to no success outcomes. These findings, consistent with Iván-Baragaño et al. [22], suggest that in MM situations, the team regaining possession only needs to bypass the opponent’s midfield and deeper defensive lines to advance towards goal. In contrast, in RA situations, the team must overcome all the opponent’s defensive lines, which complicates progression and diminishes offensive effectiveness. This aligns with the observation that a greater number of opposing defenders negatively impacts the success of an attacking action.

Regarding the type of possession or attacking approach, the results indicate that long possessions are the most effective. Closely link to this, the number of passes made also plays a crucial role in offensive success. Specifically, executing five or more passes is associated with a higher likelihood of success, whereas making only one or two passes is linked to no success. As shown in Table 3, 37.6% of possessions involving five or more passes resulted in success, compared to 26.1% that did not. Conversely, when only one or two passes were made, 40.7% of possessions ended in no success, while just 31.2% led to a successful outcome. These findings align with those of Iván-Baragaño et al. [22], who also identify the number of passes as a key indicator of offensive performance. Additionally, ball recovery had a notable impact on outcome. Consistent with the study by Scanlan et al. [45], turnovers were found to be the most effective defensive actions for regaining possession and generating goal-scoring opportunities.

Finally, the contextual variables of match outcome and period of the match influenced outcome. Winning teams demonstrated greater offensive effectiveness (48.3%, Table 3), suggesting a better ability to capitalise on attacking opportunities. This finding corroborates Iván-Baragaño et al. [22], where this factor was strongly associated with goal-scoring success in the FWWC 2023. Likewise, offensive transitions executed in the final minutes of the match (76–90) were the most successful. This supports previous research, which suggests that physical and mental fatigue towards the end of a match increases the probability of defensive errors, thereby enhancing offensive effectiveness – although this specific aspect was not directly analysed in the present study [22, 46]. However, the influence of this factor was limited, and no significant relationship was found in the bivariate analysis (p > .05).

Regarding match status, while it was not included in the final predictive model, it did exhibit a significant relationship in the bivariate analysis. Specifically, winning teams held a slight advantage in offensive effectiveness (29.1% success compared to 22.6% no success, Table 3). Nevertheless, Iván-Baragaño, et al. [22] suggest that offensive effectiveness does not significantly fluctuate depending on match status.

Regarding the model’s predictive capacity, the evaluation of the confusion matrix on 30% of the validation sample from the original dataset yielded an overall accuracy of 86%, with and 18% success rate in correctly classifying shots or goals. This represents an eightpercentage-point improvement compared to the relative frequency of these events in the original dataset. Compared to the study by Iván-Baragaño et al. [22], the predictive capacity for positive events in this model was five percentage points higher, suggesting that: (i) a larger sample enhances model training and performance, and, (ii) incorporating a greater number of features and fine-tuning hyperparameters is crucial for improving target variable prediction. However, as seen in other studies addressing imbalanced datasets in football [24, 25], the inherent complexity of the sport poses challenges in identifying consistent patterns, which limits the model’s ability to accurately predict goals or shots. In this regard, it is important to consider that goals and shots occur in approximately 2% and 10% of ball possessions, respectively, which highlights the low frequency of these actions and, consequently, the difficulty in predicting them. Nonetheless, a strong concordance was observed between the results obtained through the ShAP explainability technique and the bivariate analyses, reinforcing the utility of this approach in interpreting black-box models and facilitating the practical application of findings in the sports domain. In practical terms, the ShAP analysis highlights key predictors of offensive transition success, offering coaches evidence-based guidance for training and tactical planning. Priority should be given to transitions involving at least three penetrating passes and exploiting disorganised low defensive blocks through structured high pressing. Coordinated build-up involving multiple players, along with training under end-of-half fatigue conditions, may further enhance execution and decision-making in high-pressure scenarios.

Finally, the observation instrument developed in this study has proven to be robust and reliable for analysing dynamic offensive transitions, as 13 out of the 17 evaluated indicators showed a significant relationship with outcome. Furthermore, the consistency of these findings with previous research [22, 42–45] reinforces their external validity. However, no significant relationship was found between the general defensive approach of the opposing team and outcome. In contrast, previous studies, have demonstrated the impact of defensive pressure on offensive effectiveness [22].

This study presents a novel methodological approach by identifying offensive transition styles in elite women’s football through a clustering technique that integrates both offensive and defensive variables. The large and diverse sample, drawn from major international competitions, adds to the ecological validity of the findings. Additionally, the inclusion of model interpretability techniques (ShAP) strengthens the practical utility of the results for applied settings. However, limitations include the reliance on notational data rather than spatiotemporal tracking, which may restrict the depth of tactical analysis. Furthermore, the outcomes are sensitive to the selected features and may not fully capture in-game tactical adjustments. The exclusive focus on elite-level matches also limits the generalizability of the findings to other levels of play.

Future research should therefore explore the general defensive approach of the opposing team in greater depth, examining contextual factors that may modulate its influence and further refining our understanding of its role in offensive transitions. Additionally, optimising predictive models by incorporating new success-related criteria while eliminating those with limited impact could enhance dataset efficiency and improve model performance. The findings of this study provide crucial scientific insights into the execution of dynamic offensive transitions and offer valuable practical applications for coaches and analysts in elite women’s football.

## CONCLUSIONS

In this study, 13 of the 17 criteria analysed showed an association with the outcome of dynamic offensive transitions. Of these, the number of penetrating passes and counterattacks strategies contributing the most, as shown by ShAP value. In the same way, the opponent’s low defensive position, the MM starting interaction context, the OF recovery zone, ball recovery through turnovers, and long possessions involving five or more players contributed to the model increasing the positive output probability. Additionally, contextual variables such as match status and the period of the match also demonstrated a significant impact on offensive effectiveness. In particular, winning teams and transitions executed in the final minutes of the match were found to be more effective. Encouraging long possessions with a high number of penetrating passes can facilitate ball control and goal-scoring opportunities. Furthermore, employing high pressing to recover possession in advanced areas – particularly through turnovers – and capitalising on the opponent’s initial defensive disorganisation via counterattacks are highly effective strategies. Finally, teams should strategically manage physical exertion to sustain intensity in the final phase of the match, as offensive transitions tend to be more successful in the closing minutes of play. Regarding the trained predictive model, the application of an oversampling technique, the inclusion of a greater number of features, and the use of a larger sample enhanced its predictive capacity compared to previous studies. However, despite these improvements, the model correctly predicted a goal or shot in only one out of five instances, reflecting the inherent complexity of forecasting such events in a highly dynamic and unpredictable sport such as football. Despite everything, the application of ensemble models such as Random Forest showed an improvement in predictive capacity compared to models with greater intrinsic explainability (i.e., decision trees and binary logistic regression). Moreover, the calculation of ShAP values allowed for external interpretability of the model, reducing one of the main limitations of this model.

## Data Availability

The data that support the findings of this study are openly available in Zenodo at https://zenodo.org/records/15349140
